# Proteome-wide autoantibody screening and holistic autoantigenomic analysis unveil COVID-19 signature of autoantibody landscape

**DOI:** 10.1186/s12865-026-00826-8

**Published:** 2026-03-21

**Authors:** Kazuki M. Matsuda, Yoshiaki Kawase, Kazuhiro Iwadoh, Makoto Kurano, Yutaka Yatomi, Koh Okamoto, Kyoji Moriya, Hirohito Kotani, Teruyoshi Hisamoto, Ai Kuzumi, Takemichi Fukasawa, Asako Yoshizaki-Ogawa, Masanori Kono, Tomohisa Okamura, Hirofumi Shoda, Keishi Fujio, Kei Yamaguchi, Taishi Okumura, Chihiro Ono, Yuki Kobayashi, Ayaka Sato, Ayako Miya, Rikako Uchino, Yumi Murakami, Hiroshi Matsunaka, Hiroshi Imai, Shinichi Sato, Rudy Raymond, Ayumi Yoshizaki, Naoki Goshima

**Affiliations:** 1 Department of Dermatology, The University of Tokyo Graduate School of Medicine, 7-3-1, Hongo, Bunkyo-ku, Tokyo, 1138655 Japan; 2 Department of Computer Science, The University of Tokyo Graduate School of Information Science and Technology, Tokyo, Japan; 3 Department of Clinical Laboratory Medicine, The University of Tokyo Graduate School of Medicine, Tokyo, Japan; 4 Graduate School, International University of Health and Welfare, Tokyo, Japan; 5 Department of Infectious Diseases, The University of Tokyo Hospital, Tokyo, Japan; 6 Division of Infection Prevention and Control, Postgraduate School of Healthcare, Tokyo Healthcare University, Tokyo, Japan; 7 Department of Allergy and Rheumatology, The University of Tokyo Graduate School of Medicine, Tokyo, Japan; 8 ProteoBridge Corporation, Tokyo, Japan; 9 NOV Academic Research, TOKIWA Pharmaceutical Co., Ltd., Tokyo, Japan

**Keywords:** Autoantibody, Autoantigenomics, COVID-19, Proteome-wide autoantibody screening, Wet protein array

## Abstract

This study presents “aUToAntiBody Comprehensive Database (UT-ABCD)”, a comprehensive catalog of autoantibody profiles in 284 human individuals. The subjects include patients diagnosed with Coronavirus disease 2019 (COVID-19; *n* = 73), systemic sclerosis (SSc; *n* = 32), systemic lupus erythematosus (SLE; *n* = 60), anti-neutrophil cytoplasmic antibody-associated vasculitis (AAV; *n* = 29), atopic dermatitis (AD; *n* = 26), as well as healthy controls (HC; *n* = 64). Our investigation employs proteome-wide autoantibody screening (PWAbS) that utilizes 13,352 autoantigens displayed on wet protein arrays (WPAs). Our WPAs display human proteins synthesized in vitro utilizing a wheat germ cell-free system, maintained in a hydrated state. Our findings demonstrated significant increases in the number of IgG autoantibodies in COVID-19, SSc, SLE, AAV, and AD compared to HCs, whereas the sum of IgG autoantibody levels was significantly higher in COVID-19, SSc, and SLE. Employing machine learning, we distinguished COVID-19 cases with high accuracy based on autoantibody profiles, notably identifying antibodies against proteins encoded by *BCORP1* and *KAT2A* as highly specific to COVID-19 (specificity: 87% and 97%, respectively). Our research highlights the effectiveness of integrating PWAbS and autoantigenomics in exploring immune responses in COVID-19 and other diseases. Although external validation and functional assays were not performed, the identified reactivities represent serological biomarkers and immune signatures associated with COVID-19 and provide a comprehensive resource for future biomarker discovery.

**Supplementary Information:**

The online version contains supplementary material available at 10.1186/s12865-026-00826-8.

## Background

Coronavirus disease 2019 (COVID-19), an infectious disease caused by severe acute respiratory syndrome coronavirus 2 (SARS-CoV-2) [1], has brought a global pandemic since early 2020 with threat on human health and public safety throughout the world [2]. The pathophysiology of COVID-19 is characterized by multiple organ injuries triggered by excessive immune response [3, 4]. Cytokine storm in the lung causes acute respiratory distress syndrome, which leads to hypoxemia, respiratory failure, requirement of ventilation, and even death. One of the biggest challenges in clinical management of COVID-19 patients lies in accurately identifying and categorizing cases at higher risk of such serious clinical course. Known risk factors include older age, male gender, smoking, diabetes, obesity, hypertension, immunodeficiency, and malignancies [5].

Humoral immunity plays pivotal roles in COVID-19. Although dramatic success of mRNA vaccines and SARS-CoV-2 neutralizing monoclonal antibodies in preventing serious illnesses, accumulating evidence have suggested the vicious roles of dysregulated humoral immunity [6–10]. As well as earlier work linking anti-cytokine antibodies to mycobacterial, staphylococcal and fungal diseases [11, 12], autoantibodies against cytokines have been described in COVID-19 [13]. Especially, anti-type I interferon antibodies distinguished ~ 10% of life-threatening pneumonia and ~ 20% of deaths from COVID-19 [6–8]. A high-throughput screening by yeast display of the secretome further revealed the presence of autoantibodies against several immune factors, including chemokines [14]. In addition, autoantibodies characteristic of systemic autoimmune disorders, such as anti-phospholipid antibodies, anti-nuclear antibodies and rheumatoid factor, were reported in COVID-19 [15]. More recently, association between COVID-19 severity and autoantibodies targeting G protein-coupled receptors and renin-angiotensin system-related proteins has been reported [16].

To comprehensively understand such clinical significance of autoantibodies in human diseases including COVID-19, high-precision autoantibody measurement with a proteome-wide scale is necessary. Herein, we employed an original protein microarray technology, which includes over 13,350 proteins, for proteome-wide autoantibody screening (PWAbS) [17–19], in the serum samples derived from individual patients. Our pipeline integrates human cDNA library (HuPEX) [20], a wheat germ cell-free system for high-throughput in vitro protein synthesis [21–23], and technology for manipulating protein arrays kept in moist conditions during the entire handling process [24], namely wet protein arrays (WPAs). We have applied this method in multiple inflammatory or malignant disorders for validating its potential for illustrating the “autoantibody landscape” of human disorders, which revealed its usefulness for holistic evaluation of disease-related autoantibodies [19], developing novel biomarkers [17], and moreover, investigating unknown pathophysiology driven by autoantibodies [25].

Our aim was to demonstrate the utility of our omics-based methodology for autoantibody evaluation and data interpretation procedure, so-called “autoantigenomics,” targeting COVID-19. In 2020, Moritz et al. defined autoantigenomics as a branch of systems immunology, which holistically analyze the repertoire of autoantibodies engaging omics-based bioinformatical approaches including hierachical cluster analysis, enrichment analysis, and machine learning [26]. The concept of autoantigenomics stand on hypotheses that there might be differences in the sets of targeted antigens underlying intra-disease heterogeneity in human, which would be supported by our novel data shown below. This is attributable to the critical role that interactions between antigenic proteins and the immune system play in driving the production of disease-specific autoantibodies.

## Methods

### Human subjects

We consecutively enrolled patients administered to our institution for COVID-19 from April 2020 to April 2021. Inclusion criteria were a SARS-CoV-2 positive nasopharyngeal swab test by real-time reverse transcription-polymerase chain reaction (RT-PCR) and age > = 18 years. A Ct value of 38 cycles was used as the threshold for determining a positive result in RT-PCR. Clinical data were collected by retrospective review of electric medical records. We gathered basic patient information, symptoms, medications, histopathologic features, and laboratory findings from the closest time point from the date of serum collection. The disease severity was assessed following the Japanese guideline for managing COVID-19 patients [27]. In brief, individuals requiring intensive care or mechanical ventilation were categorized as severe COVID-19, those exhibiting hypoxemia among the remaining cases were classified as moderate COVID-19, and all other patients were considered to have mild COVID-19. Data were derived from samples collected at an “early” time point, defined as within 10 days of symptom onset. Additionally, for Fig. 5, we included samples from the same COVID-19 patients collected at a “late” time point (11–20 days after symptom onset) in 41 patients, where available. We also gathered serum samples from healthy controls (HCs) and patients with atopic dermatisis (AD), anti-neutrophil cytoplasmic antibody-associated vasculitis (AAV), systemic lupus erythematosus (SLE), and systemic sclerosis (SSc). HCs were randomly recruited from healthcare providers with no medical history, who underwent annual health checkups. Patients with AD, AAV, SLE, and SSc were recruited from individuals regularly visiting The University of Tokyo Hospital, all of whom met the diagnostic or classification criteria for their respective conditions [28–31]. This study has been approved by The University of Tokyo Ethical Committee (Approval number 0695 and 2023051G). Written informed consent has been obtained from all the participants. All methods were performed in accordance with the relevant guidelines and regulations, including the Declaration of Helsinki.

### Measurement of IgG targeting SARS-CoV-2 particles

The process of quantifying IgG antibodies that target specific SARS-CoV-2 proteins, namely the nucleocapsid protein, spike protein, and the spike protein’s receptor binding domain, was conducted as outlined previously using a commercial SARS-CoV-2 IgG kit (YHLO Biotechnology Company, Ltd., Shenzhen, China) [32, 33]. This involved an assay where serum samples were combined with magnetic beads coated with the viral proteins and a substance to prepare the samples. This mix was then washed, combined with an acridinium-conjugated anti-human IgG, and washed again. The subsequent steps included adding solutions to induce a chemiluminescent reaction, the intensity of which was measured by the iFlash3000 CLIA analyzer (YHLO Biotechnology Company, Ltd.) A threshold of 10 AU/mL was used for the detection, following the guidelines provided by the manufacturer.

### Autoantibody measurement

WPAs were arranged as previously described [17]. First, proteins were synthesized in vitro utilizing a wheat germ cell-free system from 13,352 clones of the HuPEX [20]. TRIM21 and POLR3A existed in two forms: full-length and truncated. A truncated TRIM21 construct (TRIM21(1-400)) lacking the C-terminal immunoglobulin-binding domain was used to reduce nonspecific background signals [34], while a truncated POLR3A construct (POLR3A_D) previously reported to enhance detection sensitivity was included alongside the full-length protein [35, 36]. All other proteins were full-length. Second, synthesized proteins were plotted onto glass plates (Matsunami Glass, Osaka, Japan) in an array format by the affinity between the GST-tag added to the N-terminus of each protein and glutathione modified on the plates. All autoantigens were presented in duplicate on the WPAs. The array also included mock spots as negative controls (wheat germ cell-free protein synthesis products without any cDNA) and IgG spots as positive controls (wheat germ cell-free protein synthesis products with IgG-coding cDNA). The WPAs were treated with human serum diluted by 1:333 in the reaction buffer containing 1x Synthetic block (Invitrogen), phosphate-buffered saline (PBS), and 0.1% Tween 20. Next, the WPAs were washed, and goat anti-Human IgG (H + L) Alexa Flour 647 conjugate (Thermo Fisher Scientific, San Jose, CA, USA) diluted 1000-fold was added to the WPAs and reacted for 1 h at room temperature. Finally, the WPAs were washed, air-dried, and fluorescent images were acquired using a Typhoon imaging system (Cytiva, Marlborough, MA, USA). Fluorescence images were analyzed to quantify serum levels of autoantibodies targeting each antigen, following the formula shown below:

$$\mathrm{Autoantibodylevel}\left[\mathrm{AU}\right]=\frac{{\mathrm{F}}_{\mathrm{autoantigen}}-{\mathrm{F}}_{\mathrm{negativecontrol}}}{{\mathrm{F}}_{\mathrm{positivecontrol}}-{\mathrm{F}}_{\mathrm{negativecontrol}}}\times100$$

$$AU:\;\text{arbitrary unit}$$

$$F_{negative control}:\;\text{fluorescent intensity of negative control spot}$$

$$F_{autoantigen}:\;\text{fluorescent intensity of autoantigen spot}$$

$$F_{positive control}:\;\text{fluorescent intensity of positive control spot}$$

Potential batch effects were considered by using a standardized WPA platform with consistent reagents and procedures, normalization to internal negative and positive controls, and by processing samples from each disease condition across multiple batches, as each condition included more samples than the batch size (*n* = 3), thereby avoiding confounding between condition and batch. The linearity of measurement has been assured within the range of 0–100 AU based on standard IgG dilution series spotted on the WPAs. Signals exceeding this range were estimated by extrapolation. When there was a significant discrepancy between the results of duplicate spots, we examined the raw fluorescence images and excluded abnormal signals, such as noise generated by dust particles. SAL was defined as the sum of serum levels for all 13,352 autoantibodies tested in our study.

### Machine learning

We trained supervised machine learning models for classification tasks using simple linear regression, Ridge regression, logistic regression with normalization, logistic regression with standardization, SVM with normalization, SVM with standardization, and XGBoost. These models were implemented using the scikit-learn library (https://scikit-learn.org/) in Python (v.3.11.11) and trained within a Jupyter Notebook environment. Hyperparameter tuning was conducted using Optuna (https://optuna.org/).

For simple linear regression, Ridge regression, and logistic regression, we used the “sklearn.linear_model” module. For Ridge regression, the hyperparameter “alpha” was searched between 0.0001 and 10.0 on a logarithmic scale. For SVM, we used the “sklearn.svm” module. For logistic regression and SVM, we performed standardization or normalization for each autoantibody using the “StandardScaler” and “MinMaxScaler“ from the “sklearn_preprocessing” module. For XGBoost, we used the “xgboost” module and performed hyperparameter tuning over the following ranges: “n_estimators” (100–1000), “max_depth” (3–9), “learning_rate” (1e-3–1e-1) on a logarithmic scale, “subsample” (0.6–1.0), “colsample_bytree” (0.6–1.0), “min_child_weight” (1–10), “reg_lambda” (1e-8–10.0) on a logarithmic scale, and “reg_alpha” (1e-8–10.0) on a logarithmic scale.

### Performance metrics

We performed the 10-fold cross-validation as follows: first, we shuffled and split the data into 10 equally sized parts, selecting one of these parts as validation data while the remaining 9 parts as training data. We then repeated this procedure 10 times, selecting a different part as the validation data. Model performance on the testing set was evaluated using the following metrics:

- Accuracy: $$\frac{TP+TN}{TP+FP+TN+FN}$$  
- Precision: $$\frac{TP}{TP+FP}$$
- Recall: $$\frac{TP}{TP+FN}$$
- F1-score: $$\frac{2\;\times\;TP}{2\;\times\;TP+FP+FN}$$  
- Matthew’s Correlation Coefficient (MCC): $\frac{TP\;\times\;TN-FP\;\times\;FN}{\sqrt{(TP+FP)(TP+FN)(TN+FP)(TN+FN)}}$  

TP: true positive.

FP: false positive.

TN: true negative.

FN: false negative.

### Feature importance scores and feature selection

The feature importance was indicated by their mean absolute coefficient in the 10-fold cross validation in simple linear regression, Ridge regression, and logistic regression. As for SVM and XGBoost, the feature importance was represented by F-scores, meaning the average number of splits by each feature over the cross-validation.

Model features were directly derived from proteome-wide autoantibody measurements obtained by WPAs. For each serum sample, IgG binding to 13,352 human proteins was quantified, generating a continuous numeric vector of autoantibody intensities. Each subject was therefore represented by a 13,352-dimensional feature vector, with each dimension corresponding to serum reactivity against a specific autoantigen. When constructing minimum feature models, we selected only the top 1–5 features ranked by F-scores for final model training in each cross-validation iteration. Sensitivity analyses were performed by including age and sex as additional features, excluding truncated autoantigens (TRIM21(1–400) and POLR3A_D), or excluding critically ill patients (severe COVID-19 cases).

### Enzyme-linked immunosorbent assay

Two 96-well antigen-binding plates were prepared: one for antigen coating and another for negative control. Each well was initially filled with 50 µL of phosphate-buffered saline (PBS). After incubation for 5 min at room temperature (RT), the liquid was removed. A solution of recombinant KAT2A (MyBioSource, San Diego, CA, USA) expressed in a baculovirus-insect cell system or recombinant BCOR (OriGene Technologies, Rockville, MD, USA) expressed in HEK293 cells was prepared at a concentration of 3 µg/mL in PBS. Subsequently, 30 µL of the antigen solution was added to each well of the antigen coating plate, while 30 µL of PBS alone was added to the wells of the negative control plate. The plates were then incubated at 300 rpm for 1.5 h at RT to facilitate antigen binding. After incubation, the plates were washed three times with 120 µL of PBST (PBS containing 0.1% Tween-20) per well. Blocking was performed by adding 100 µL of blocking buffer (0.5% skim milk in PBST) to each well. The plates were sealed with transparent adhesive film and incubated at 300 rpm for 1 h at RT. Following blocking, adjusted serum reaction solutions were prepared and pipetted into the plates, with 50 µL added to each well. The plates were sealed with aluminum adhesive film and incubated at 860 rpm for 1 h at RT. The plates were washed three times with 120 µL of TBST (Tris-buffered saline containing 0.1% Tween-20). Subsequently, 50 µL of secondary antibody solution, goat anti-Human IgG (H + L) Alexa Flour 647 conjugate (Thermo Fisher Scientific) diluted 1:1000 in TBST, was added to each well. The plates were again sealed and incubated for 1 h at RT. After the secondary antibody reaction, the plates were washed three times with 120 µL of TBST. Then, 50 µL of TBST was added to each well. Fluorescence signals were scanned using a Typhoon imaging system (Cytiva). The serum autoantibody levels were quantified by calculating the difference in fluorescent signals between the antigen-coated plate and the negative control plate.

### Statistical analysis

Two-group comparisons were performed using two-sided Fisher’s exact test for categorical variables and two-sided Mann-Whitney U test for continuous variables. P-value of < 0.05 was considered statistically significant. Differentially elevated autoantibodies were defined as more than 2-fold changes in the serum levels with a false discovery rate (FDR) < 0.05, adjusted using the Benjamini-Hochberg method. Gene Ontology Analysis using web-based tools targeted the list of the entry clones coding the differentially highlighted autoantigens was performed for gene-list enrichment analysis, gene-disease association analysis, and transcriptional regulatory network analysis with Metascape [37]. As a reference, we utilized the list of all the genes included in our cDNA library used for WPA manipulation. Other data analyses and presentations were conducted using Stata IC/15.0 (StataCorp, TX, USA).

### Data visualization

Box plots, scatter plots, hierarchical clustering and correlation matrix were visualized by using R (v4.2.1). Box plots were defined as follows: the middle line corresponds to the median; the lower and upper hinges correspond to the first and third quartiles; the upper whisker extends from the hinge to the largest value no further than 1.5 times the interquartile range (IQR) from the hinge; and the lower whisker extends from the hinge to the smallest value at most 1.5 times the IQR of the hinge.

## Results

### Overview

**Fig. 1:**
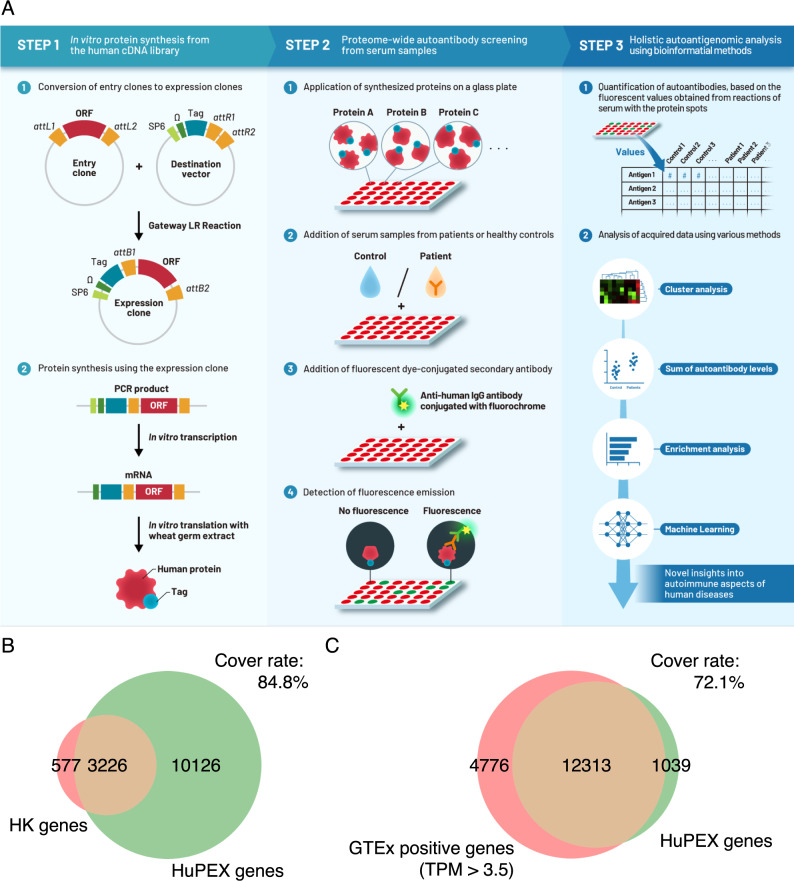
Overview of PWAbS methodology. **A** Scheme of PWAbS pipeline. In the first step, proteins were synthesized in vitro from the proteome-wide human cDNA library (HuPEX). Promotors (P), Enhancers (E), and FLAG-GST tags were fused to open reading frames of the expression clones by Gateway LR reaction. After polymerase chain reaction amplification and in vitro transcription, translation was performed using the wheat germ cell-free synthesis system. In the second step, we prepared WPAs by plotting synthesized proteins onto glass slides in an array format. WPAs were treated with serum samples derived from diseased patients or HCs. Autoantibodies were detected by fluorochrome-conjugated anti-human IgG Ab. In the third step, autoantibody quantification was performed based on the fluorescent values. Analysis of acquired high-dimensional autoantibody profiles was conducted by multiple omics-based approaches. ORF: open reading frame. **B** A venn diagram shows our WPA covers 84.8% of human housekeeping genes. **C** A venn diagram illustrates our WPA covers 72.1% of expressed human genes, based on genes expressed at more than 3.5 TPM in the GTEx V8, a bulk RNA-seq data from 52 human tissues and two cell lines

We recruited 284 human subjects in total. The mean age was 50 ± 20 years in mild COVID-19 (*n* = 22), 67 ± 14 years in moderate/severe COVID-19 (*n* = 51), 36 ± 15 years in AD (*n* = 26), 70 ± 14 years in AAV (*n* = 29), 49 ± 14 years in SLE (*n* = 60), 59 ± 13 years in SSc (*n* = 32), and 40 ± 15 years in HCs (*n* = 64). The proportion of males was 64% in mild COVID-19, 65% in moderate/severe COVID-19, 62% in AD, 41% in AAV, 7% in SLE, 19% in SSc, and 50% in HCs (Extended Table 1). For each individual serum, PWAbS was performed focusing on IgG autoantibodies (Fig. 1A). Our WPA included 84.8% of previously reported housekeeping genes (Fig. 1B) [38]. Based on genes expressed at more than 3.5 transcripts per million (TPM) in the Genotype-Tissue Expression (GTEx) V8 [39], our WPA covered 72.1% of expressed human genes (Fig. 1C).

The digest of the results is available as “aUToAntiBody Comprehensive Database (UT-ABCD)” at http://www.ut-abcd.org. We found that sum of autoantibody levels (SAL), defined as the sum of all quantified values for the 13,352 displayed autoantigens, was significantly elevated in patients with COVID-19 (median [interquartile range]: 4418 [3325–5392] AU), SSc (5784 [3960–7338] AU), or SLE (5963 [4543–7386] AU), compared to HCs (3014 [2289–3851] AU), while there was no statistically significant difference in SAL between AD (3664 [2419–5451] AU) or AAV (3842 [3281–4547] AU) patients and HCs (Extended Fig. 1A). This tendency was consistent across both gender and age groups. (Extended Fig. 1B). The proportion of measurements exceeding 100 AU in each condition was as follows: HC: 0.36%, COVID-19: 0.26%, AD: 0.29%, AAV: 0.21%, SSc: 1.43%, and SLE: 1.30%. These findings suggest that the observed pattern was not substantially confounded by age or sex, and that the impact of data extrapolation was limited.

### Threshold determination and positivity counts

**Fig. 2:**
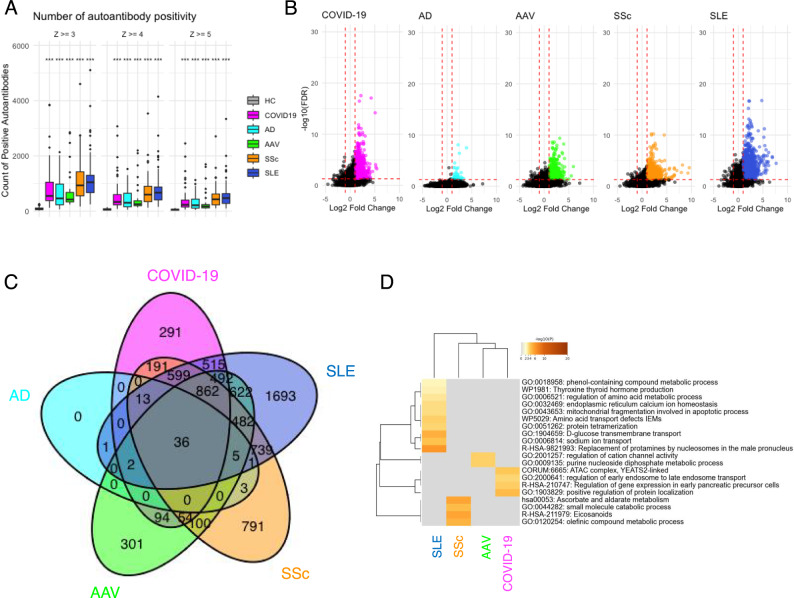
Identification of disease-specific autoantibodies. **A** Box plots that show the count of autoantibody positivity in each individual, defined by a threshold of Z-score > = 3, 4, or 5 in healthy controls (HCs). ***: *P* < 0.001. P-values were calculated by two-sided Mann Whitney U test compared to HCs. **B** Volcano plots that illustrate differentially elevated autoantibodies within each condition compared to HCs. Red horizontal dash lines indicate false discovery rate (FDR) = 0.05. FDR was calculated by two-sided Mann Whitney U test with Benjamini-Hochberg correction compared to HCs. Red vertical dash lines indicate Log2 Fold Change (Disease/HC) = ± 1. **C** Venn diagram that demonstrates the subsumptions among disease-specific autoantibodies. **D** Heat map that shows the result of enrichment analysis targeting the genes responsible for the proteins targeted by such disease-specific autoantibodies. P values were calculated utilizing the list of genes included in our cDNA library used for WPA manipulation as a reference

To establish the threshold for determining serum positivity of each autoantibody, Z-scores were calculated based on the mean and standard deviation values among healthy controls (HCs). The percentage of Z-scores in HCs above each threshold was 2.41% for Z = 3, 1.16% for Z = 4, and 0.55% for Z = 5 (Extended Fig. 1C and D, and 1E). Consequently, a threshold of Z > = 4 was selected as a stringent criterion to minimize false positives while maintaining detection sensitivity. The number of positive autoantibodies was then counted for each individual and compared across different conditions (Fig. 2A). This analysis demonstrated a significant increase in the number of autoantibodies in patients with COVID-19, AD, AAV, SLE, and SSc compared to HCs. Sensitivity analyses using alternative cutoffs (Z > = 3 and Z > = 5) confirmed that the significant increase in autoantibody counts across all disease groups compared to HCs remained statistically consistent regardless of the threshold chosen.

### Identification of disease-specific autoantibodies

We identified distinct sets of autoantibodies that showed a more than twofold significant increase in each disease condition relative to HCs (Fig. 2B). Notably, certain autoantibodies were unique to each disease (Fig. 2C). Gene ontology analysis linked to the genes responsible for the proteins targeted by such disease-specific autoantibodies pointed to shared biological functions, with a focus on viral infection pathways and cytokine signaling in immune system, in COVID-19, SLE, and AAV (Fig. 2D). Holistic analysis of autoantibodies targeting cytokines, or their receptors displayed on our WPAs revealed that strong positivity for autoantibodies targeting type 1 interferon was specifically observed in COVID-19 patients, while weak positivity was seen in SLE patients (Extended Fig. 2), in line with previous studies [6, 40].

### Selection of machine learning frameworks

To further investigate the association between autoantibody profiles and COVID-19, we adopted a machine learning approach. We tested seven different methods to differentiate COVID-19 cases from the others: simple linear regression, Ridge regression, logistic regression with data normalization, logistic regression with data standardization, support vector machine (SVM) with data normalization, SVM with data standardization, and extremely gradient boosting decision trees (XGBoost). The distribution of the normalized values across conditions is shown in Extended Fig. 3. As a result, XGBoost showed the highest value of accuracy, recall, F1-score, and Matthew’s Correlation Coefficient (MCC) for distinction of COVID-19 cases from the others (Extended Table 2). Consequently, we opted to focus on this method for our subsequent analysis.

### Performance of XGBoost

In our subsequent analysis using the entire dataset, we experimented with binary (COVID-19 vs. others), ternary (mild COVID-19 vs. moderate/severe COVID-19 vs. others), and multiclass (mild COVID-19 vs. moderate/severe COVID-19 vs. AAV vs. AD vs. SSc vs. SLE vs. HCs) classifications through XGBoost. The binary and ternary tasks were designed to test whether COVID-19 and its severity exhibit disease-specific autoantibody signatures against a heterogeneous immune background, whereas the multiclass task aimed to assess disease-specific discrimination across individual conditions.

**Fig. 3:**
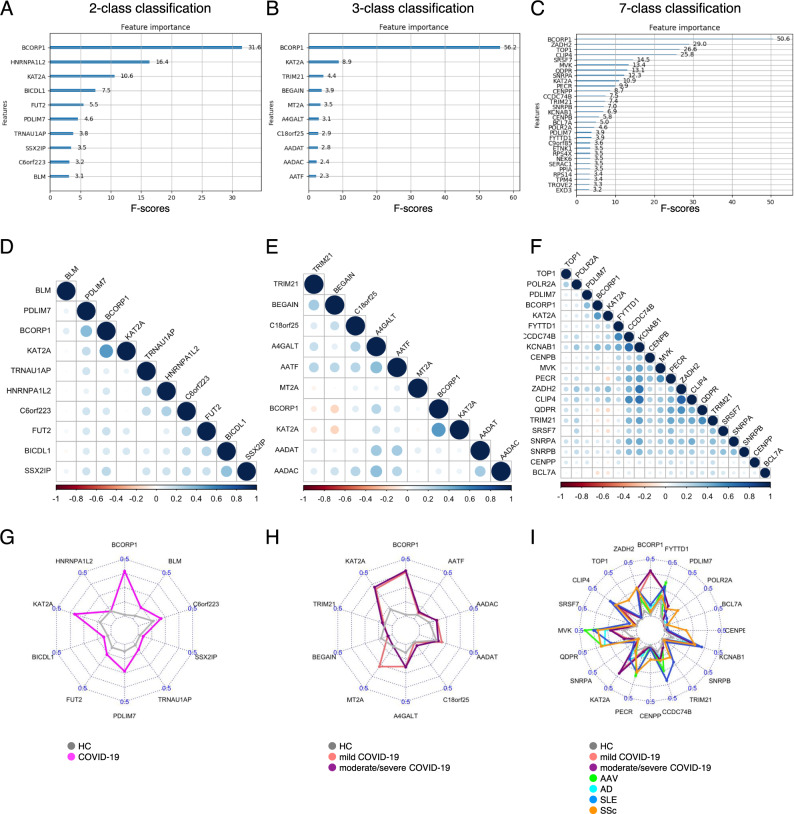
Autoantibodies highlighted in each machine learning model. Autoantibodies that are mostly highlighted according to feature importance in two-class (**A**), three-class (**B**), and multi-class (**C**) classifications. The feature importance is represented by F-scores, meaning the average number of splits by each feature over the cross-validation. Correlograms depict correlations, showcasing the connection between the highest-ranked autoantibodies in two-class (**D**), three-class (**E**), and multi-class (**F**) classifications. The correlation strength is denoted by Spearman’s rho on the color scale. Circle sizes represent the significance of the p-values, with only those with *P* < 0.05 being displayed. Radar charts present the average normalized quantities of the most important autoantibodies in each model, with line colors distinguishing between different disease categories in two-class (**G**), three-class (**H**), and multi-class (**I**) classifications

The models achieved high accuracy in both binary and ternary classifications and showed significantly better outcomes than chance in the complex seven-class classification (Extended Table 3). The most significant autoantibodies identified across all models are depicted in Fig. 3A and B, and 3C, with autoantibodies against translational products from *BCL6 Corepressor Pseudogene 1* (*BCORP1*) emerging as a top feature in every model. Similarly, antibodies against K-Acetyltransferase 2 A (KAT2A) were consistently prominent. Notably, Anti-BCORP1 and anti-KAT2A Abs were highlighted as important items in all the candidate machine learning methods tested (Extended Fig. 4A, B, C, D and E, and 4F). There was a correlation between anti-BCORP1 Abs and anti-KAT2A Abs as illustrated in Fig. 3D and E, and 3F. Remarkably, established serum markers for SSc and SLE, such as anti-topoisomerase 1 (TOP1) Abs, anti-centromere protein-B (CENPB) Abs, anti-tripartite motif-containing protein 21 (TRIM21) Abs, anti-small nuclear ribonucleoprotein polypeptide (SNRP)-A Abs, and anti-SNRPB Abs, were also identified. The visualization of mean serum levels of these prominent markers through spider charts revealed distinctive patterns across the different conditions (Fig. 3G and H, and 3I).

We also attempted to integrate clinical features, specifically sex and age, into the classifiers. As a result, while age ranked second in the 3-class classification task and in the fourth in the 7-class classification task (Extended Fig. 4G and H, and 4I), it did not lead to improvement in overall performance across all classification tasks (Extended Table 3). Moreover, we conducted sensitivity analyses by excluding truncated autoantigens (POLR3A_D and TRIM21(1–400)) or removing critically ill patients. The primary classification signatures identified by the machine learning models, including those for COVID-19, remained robust. In summary, the classification framework showed strong robustness, with minimal contribution from basic clinical covariates and stable performance across multiple sensitivity analyses.

### Minimum feature model

We evaluated the effectiveness of key features identified by machine learning algorithms for COVID-19 classification by training an XGBoost model during each iteration of cross-validation, using the top 1 to 5 features highlighted in the previous 2-class classification task (Extended Fig. 5). Our analysis showed that even a single feature, BCORP1, achieves area under the receiver-operator characteristics curve (ROC-AUC) exceeding 0.85, demonstrating strong discriminative power. While incorporating additional features enhanced some performance metrics, we observed a plateau beyond three features, suggesting that minimal models can still achieve high clinical relevance.

To further validate these findings, we trained a logistic regression model using the top two and top five features (Extended Table 4). Two-feature model (BCORP1 and KAT2A) achieved an AUC of 0.913, confirming that these markers are highly informative for COVID-19 classification. Five-feature model (BCORP1, KAT2A, RPS4X, BEGAIN, and TRIM21) improved accuracy, recall, precision, and F1-score. However, the ROC-AUC remained like that of the two-feature model. These results indicate that BCORP1 and KAT2A are key features for distinguishing COVID-19 cases, and our machine-learning approach is effective in identifying important biomarkers.

### Clinical relevance of autoantibodies

**Fig. 4:**
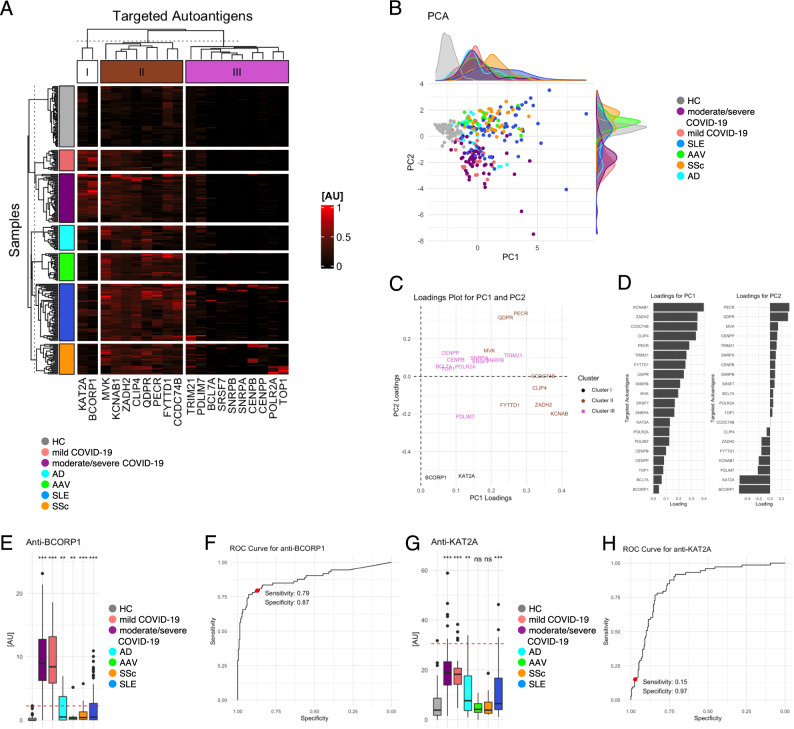
COVID-19 signature of autoantibody landscape. **A** The heatmap’s columns display the serum autoantibody concentrations highlighted in the multi-class classification using XGBoost. **B** PCA graph plots individual participants as points, with color coding to differentiate among various disease classes. **C** The loading diagram illustrates the contributions to PC1 and PC2, with I, II, and III marking the clusters defined in (A). **D **The bar graphs show the loadings of each autoantibody on PC1 and PC2. **E** A box plot presents the serum levels of anti-BCORP1 Abs in the subjects. **: *P* < 0.01, ***: *P* < 0.001. P-values were calculated by two-sided Mann Whitney U test compared to HCs. The red horizontal dashed line indicates Z-score = 4 in HCs. **F** A ROC curve illustrates the sensitivity and specificity for COVID-19 identification by serum levels of anti-BCORP1 Abs. The red dot marks the cutoff value set at Z-score = 4 in HCs. **G** A box plot indicates the serum levels of anti-KAT2A Abs in the subjects. ns: *P* > 0.05, **: *P* < 0.01, ***: *P* < 0.001. P-values were calculated by two-sided Mann Whitney U test compared to HCs. The red horizontal dashed line indicates Z-score = 4 in HCs. **H** A ROC curve illustrates the sensitivity and specificity for COVID-19 identification by serum levels of anti-KAT2A Abs. The red dot marks the cutoff value set at Z-score = 4 in HCs

The serum levels of the top 20 autoantibodies highlighted through multi-class classification through XGBoost for each participant were depicted in a heatmap (Fig. 4A). Hierarchical clustering identified three unique groups of autoantibodies: cluster I, which included two autoantibodies highly specific to COVID-19 (anti-BCORP1 and anti-KAT2A Abs); cluster II, comprising autoantibodies that are commonly elevated across various conditions; and cluster III, involving well-established biomarkers for SSc and SLE. Principal component analysis (PCA) effectively distinguished between seven categories (Fig. 4B), particularly using principal component (PC) 2 as an indicator for COVID-19 (Fig. 4C). Correspondingly, antibodies against BCORP1 and KAT2A constituted the predominant contribution to PC2 (Fig. 4D).

To validate the PWAbS results using WPAs in COVID-19 patients, we performed enzyme-linked immunosorbent assays (ELISA) to quantify serum levels of anti-KAT2A antibodies, employing recombinant KAT2A produced in a Baculovirus expression system. The analysis revealed a statistically significant positive correlation between the results obtained from PWAbS and ELISA (Extended Fig. 6A). In contrast, ELISA using recombinant BCL2 co-repressor (BCOR), which shares approximately 99% nucleotide sequence similarity with BCORP1 and was synthesized in HEK293 cells, showed no correlation with the serum levels of anti-BCORP1 antibodies measured by WPAs (Extended Fig. 6B).

Serum levels of anti-BCORP1 were the highest in COVID-19 among the examined conditions (Fig. 4E). This trend was consistent among both sex and age groups (Extended Fig. 6C). When the cutoff value was set at the mean + 4SD (Z-scores >= 4) in healthy controls (HCs), only one individual in the HC group showed serum positivity for BCORP1, compared to 61 patients with COVID-19 (*P* < 0.001, two-sided Fisher’s exact test). Receiver operating characteristic (ROC) curve analysis demonstrated a sensitivity of 79% and a specificity of 87% (Fig. 4F). For anti-KAT2A antibodies, serum levels were highest in COVID-19 patients across all age and sex groups (Fig. 4G and Extended Fig. 6D). With the cutoff set at Z-scores > = 4 in HCs, only one HC individual showed serum positivity for KAT2A, while 16 COVID-19 patients tested positive (*P* = 0.0045, two-sided Fisher’s exact test). ROC curve analysis indicated a sensitivity of 15% and a specificity of 97% (Fig. 4H). We also explored the link between COVID-19 clinical outcomes and the presence of anti-BCORP1 or anti-KAT2A Abs, but no significant association was found (Extended Tables 5 and 6).

### Time course of autoantibody levels during COVID-19

Finally, we enhanced our analysis by including “late” time point samples. The data presented thus far derive exclusively from “early” time points, defined as within 10 days of symptom onset. To conduct a longitudinal evaluation of the humoral immune response, we incorporated paired serum samples from 41 individuals, including both “early” time points and “late” time points, defined as 11–20 days after symptom onset. These samples were used for PWAbS and for assessing IgG levels against SARS-CoV-2 particles: the nucleocapsid protein (N), spike protein (S), and the receptor binding domain (RBD) of S. This analysis was performed to assess whether any autoantibody responses exhibited temporal patterns similar to antiviral IgG, which would suggest potential cross-reactivity or bystander immune activation driven by viral antigens.

**Fig. 5:**
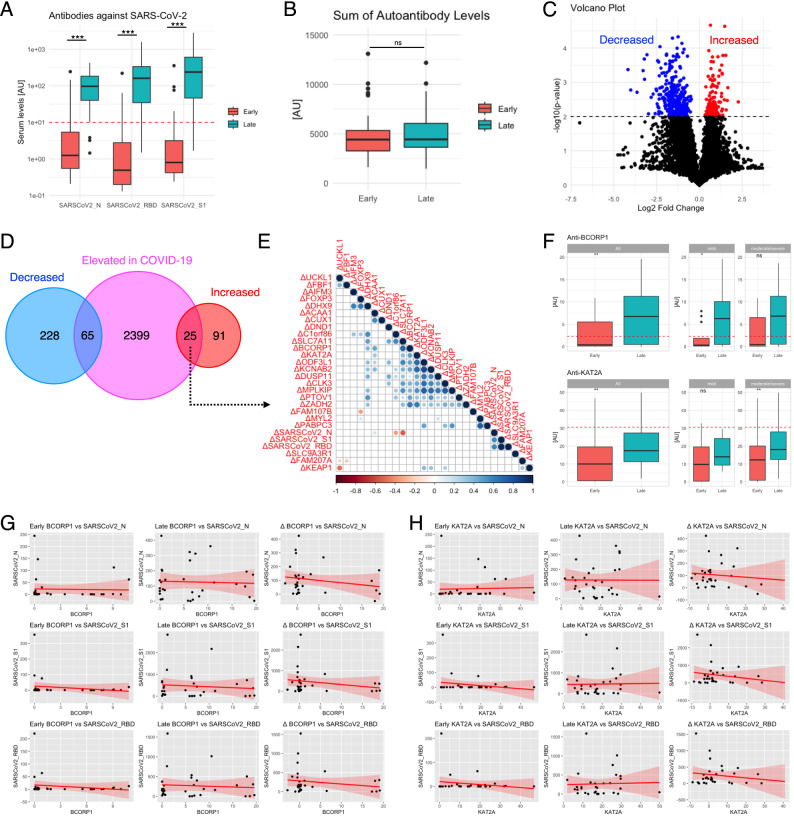
Longitudinal change of humoral immune response in COVID-19. **A** This box plot outlines the serum concentrations of IgG antibodies against N, S, or RBD in patients with COVID-19 at two intervals: “early” signifies within 10 days of symptom onset, and “late” refers to 11–20 days after symptoms appear. A red dashed line marks the threshold for a positive test result. ***: *P* < 0.001. P-values were calculated by two-sided Wilcoxon singed rank test. **B** These box plots demonstrate the SAL in patients with COVID-19 during the “early” and “late” time points. ns: *P* > 0.05. P-values were calculated by two-sided Wilcoxon singed rank test. **C** The volcano plot highlights biomarkers that are significantly increased (in red) or decreased (in blue) over time in patients with COVID-19. **D** A Venn diagram illustrates the overlap between autoantibodies that changed over time and those specific to COVID-19. **E** The correlogram visualizes the relationships between the overlapping autoantibodies that either increased over time or are specific to COVID-19, with the color scale indicating the strength of correlation according to Spearman’s rho and circle sizes depicting the significance of p-values, focusing on those with *P* < 0.05. **F** These box plots depict the time-based evolution of serum levels of anti-BCORP1 and anti-KAT2A antibodies in COVID-19 patients, categorized by disease severity. The red horizontal dashed lines indicate Z-scores = 4 in HCs. ns: *P* > 0.05, *: *P* < 0.05, **: *P* < 0.01. P-values were calculated by two-sided Wilcoxon singed rank test. **G H** The scatter plots illustrate the correlation between serum levels of anti-BCORP or anti-KAT2A Abs and IgG antibodies against the COVID-19 components at different time points, as well as their progression over time. The red lines and the surrounding shaded areas indicate the regression line and the 95% confidence interval, respectively

Consistent with our prior findings [32], early timepoint samples showed IgG against N, S, and RBD in only a small part of the patients, with a marked increase in most patients by the late timepoint (Fig. 5A). To the contrary, SAL remained unchanged over time (Fig. 5B). Further exploration revealed a significant decrease in 293 autoantibodies and increase in 116 autoantibodies over the course of the infection, including those targeting BCORP1 and KAT2A (Fig. 5C and D). There was no observed correlation between these autoantibodies and IgG levels against N, S, and RBD (Fig. 5E). Notably, both anti-BCORP1 and anti-KAT2A Ab levels rose over time, especially in mild COVID-19 group for anti-BCORP1 and in moderate/severe COVID-19 group for anti-KAT2A (Fig. 5F). Additional analysis did not find any correlation between these two autoantibodies and IgG targeting N, S, and RBD at either timepoint or their progression over time (Fig. 5G and H). This observation suggests that autoantibodies to BCORP1 and KAT2A were unlikely to arise from direct cross-reactivity with SARS-CoV-2 antigens.

## Discussion

Herein we conducted PWAbS subjecting serum samples derived from HCs and patients with COVID-19, AD, AAV, SLE, and SSc (Fig. 1A). We demonstrated that our PWAbS methodology enables us to identify disease-specific autoantibodies (Fig. 2A and B, and 2C), which demonstrate the distinct distribution of autoantibodies and common biological processes among different conditions (Fig. 2D and E, and 2F). The contrast in autoantibody profiles was accentuated through the application of a machine learning approach, particularly leveraging the XGBoost framework (Figs. 3 and 4). We also investigated the longitudinal change of autoantibody profiles along with the time course of COVID-19 and its correlation with emergence of antibodies targeting COVID-19 particles (Fig. 5). Collectively, these results supported our hypothesis that the combination of PWAbS and omics-based bioinformatic methodologies is adaptable to human disorders including COVID-19. Additionally, our findings in this study as well as our previous works provide a comprehensive catalog of autoantibody profiles in various diseases, utilizing distinct autoantibody patterns measured by WPAs [17, 19].

Unlike secretome-focused platforms such as Rapid Extracellular Antigen Profiling (REAP) [9, 10], which are optimized for detecting potentially functional antibodies against extracellular targets, PWAbS enables the assessment of intracellular autoantigens that serve as key disease-defining biomarkers in systemic autoimmune diseases such as SSc and SLE. We also compared our findings with those of Jernbom et al. [41], who profiled new-onset autoantibodies in individuals with COVID-19 using protein fragment-based planar arrays. Their study leveraged a powerful longitudinal design, including pre-infection baseline samples, which enabled precise identification of newly emerging autoantibodies following SARS-CoV-2 exposure. Although the exact expression system used was not specified, the use of protein fragments likely favored detection of linear epitopes but may have limited the capacity to capture conformational epitopes. In contrast, our approach utilized full-length, hydrated proteins synthesized in a wheat germ cell-free system, which may better preserve native-like folding and facilitate detection of structurally complex epitopes. Despite these methodological differences, both studies independently demonstrated he persistence of autoantibodies long after acute infection and marked inter-individual heterogeneity in autoantibody profiles. These shared findings reinforce the robustness of virus-induced autoimmunity as a consistent immunological feature of COVID-19.

The machine learning-based approach has discovered that the presence of anti-KAT2A antibodies is highly specific to COVID-19 (Fig. 4G and H). The levels of anti-KAT2A Abs increased over the course of COVID-19 but were not correlated to antibodies targeting SARS-CoV-2 particles (Fig. 5H). This observation indicates that autoantibodies to KAT2A emerge because of autoantigen exposure due to tissue damage triggered by COVID-19, not as a reflection of cross-reaction between SARS-CoV-2 virions. Intriguingly, the results of ELISA utilizing recombinant KAT2A produced in a Baculovirus expression system showed statistically significant positive correlation with our PWAbS results (Extended Fig. 6A), supporting the validity of our method for quantifying autoantibodies across different protein synthesis systems.

Anti-BCORP1 reactivity was among the most prominent features distinguishing COVID-19 sera from other conditions (Fig. 4E and F). *BCORP1* is annotated as a Y-chromosome pseudogene, and although multiple transcriptomic studies have reported *BCORP1*-derived RNA sequences [42, 43], there is currently no direct biochemical evidence that it produces a stable protein product. While public databases such as PaxDb and STRING contain entries suggesting possible protein abundance or interaction profiles [44, 45], these inferences are largely indirect and do not constitute experimental validation of BCORP1 as a bona fide protein-coding gene.

Moreover, anti-BCORP1 reactivity was detected in both male and female samples, although *BCORP1* is annotated as a Y-chromosome pseudogene. This observation, together with the lack of correlation between anti-BCORP1 levels and IgG against SARS-CoV-2 virions over time (Fig. 5G), suggests that the observed signal is unlikely to reflect direct molecular mimicry with viral antigens. We further examined potential cross-reactivity with BCOR, the highly homologous X-chromosomal counterpart of BCORP1 (> 99% nucleotide identity), using recombinant BCOR expressed in HEK293 cells. However, ELISA measurements of anti-BCOR antibodies did not correlate with PWAbS-based anti-BCORP1 signals (Extended Fig. 6B), indicating that the array signal is not simply explained by recognition of BCOR in this assay format.

Taken together, these findings indicate that anti-BCORP1 reactivity should be interpreted with appropriate caution. At present, it is best regarded as a high-performing serological feature identified by PWAbS and machine learning, rather than definitive evidence of a biologically functional BCORP1 protein. The true antigen(s) underlying this signal may involve epitope-, conformation-, or cross-reactivity–dependent effects of the array-expressed product, and further molecular and proteomic validation will be required to determine its biological nature. 

The limitation of our present study includes its retrospective design, a relatively small number of the subjects, and the limited spectrum of disease conditions included. The demographic differences, such as sex and age, between COVID-19 patients and other groups, including HCs, raise concerns about potential confounding, as previous studies have linked age and sex to autoantibody profiles [46–48]. Despite our stratified analyses by age and sex (Extended Figs. 1 and 3), residual demographic confounding cannot be fully excluded in this retrospective cohort. Additionally, it is important to note that critically ill patients were included only in the COVID-19 group, and elevated autoantibodies have been reported in critically ill individuals without COVID-19 [49]. Although we conducted sensitivity analyses excluding severe COVID-19 cases (Extended Table 3), the lack of severity-matched non-COVID-19 critically ill controls remains a limitation. Moreover, we could not distinguish whether the autoantibodies found in our measurement were predisposed before COVID-19 or newly appeared after infection. Furthermore, our current study did not include neutralization assays, functional readouts, or in vivo modeling to assess the biological or pathogenic effects of the detected autoantibodies. To address these gaps, our future efforts will focus on (1) accessing population-based cohorts with paired pre- and post-infection samples, (2) recruiting sex-, age-, and severity-matched controls across a broader range of disease conditions, (3) conducting functional validation of antibody-target interactions, and (4) evaluating pathogenicity in animal models.

From a technical perspective, the WPA platform uses a wheat germ cell-free expression system that lacks post-translational glycosylation, which may limit detection of autoantibodies recognizing glyco-epitopes. While this is an inherent limitation, the platform is designed for high-throughput discovery using hydrated proteins and has demonstrated practical utility for broad autoantibody screening in previous studies. In addition, fluorescence signals were linear within 0–100 AU, with higher values estimated by extrapolation; such high-intensity signals were infrequent and are unlikely to materially affect group-level comparisons, because the proportion of measurements exceeding 100 AU was relatively low across all conditions and followed a trend similar to SAL (Extended Fig. 1A). Another technical limitation of our study concerns immunoglobulin isotype coverage, as the PWAbS wet protein array is probed with a single fluorophore-conjugated secondary antibody and therefore was restricted to IgG detection in this study, precluding simultaneous reliable measurement of other isotypes (e.g., IgM or IgA).

For ELISA-based validation, we used commercially available full-length recombinant proteins expressed in a baculovirus-insect cell system (for KAT2A) and HEK293 cells (for BCOR), selected for their purity and compatibility with immunoassays. While these proteins do not exactly replicate the conditions of antigen presentation on the WPA—including differences in expression system, folding, and post-translational modifications—they provided a practical means for orthogonal validation. The observed correlation for KAT2A, but not for BCOR, underscores the need for antigen-matched and context-appropriate systems in future functional studies.

From a machine learning perspective, an important limitation of this study is the very high feature-to-sample ratio, with 13,352 autoantibody measurements and a relatively small number of subjects, together with the absence of an independent external validation cohort, which precludes claims of immediate diagnostic or predictive utility. Accordingly, the machine-learning framework was used primarily for feature discovery and biomarker identification rather than for clinical classification. To reduce the risk of overfitting, we developed minimum-feature models using only the top 1–5 autoantibodies, which showed stable cross-validation performance and consistently selected key features, including anti-BCORP1 and anti-KAT2A Abs. Although these reduced models were robust, external validation in independent cohorts remains essential to establish generalizability and clinical relevance.

## Conclusion

Our study highlights the utility of PWAbS combined with bioinformatics-based autoantigenomics in identifying disease-specific autoantibody signatures. The findings not only provide insights into the unique autoantibody profiles of COVID-19 but also underscore the potential of this approach in understanding immune dysregulation across various diseases. By leveraging machine learning, we identified anti-BCORP1 and anti-KAT2A antibodies as highly specific markers for COVID-19. Validation of these findings in external cohorts will be crucial to confirm their generalizability and clinical utility, as well as to further explore the functional roles of these autoantibodies in disease mechanisms.

## Supplementary Information

Supplementary Material 1.

Supplementary Material 2.

Supplementary Material 3.

Supplementary Material 4.

Supplementary Material 5.

Supplementary Material 6.

Supplementary Material 7.

Supplementary Material 8.

Supplementary Material 9.

Supplementary Material 10.

Supplementary Material 11.

Supplementary Material 12.

Supplementary Material 13.

## Data Availability

The digest of the results is available as “aUToAntiBody Comprehensive Database (UT-ABCD)” at http://www.ut-abcd.org. The full dataset is available upon reasonable request to the corresponding author, in compliance with ethical guidelines and participant privacy considerations. All the scripts are available at https://github.com/mkazukikom/COV19ML.

## Abbreviations

AAV: Anti-neutrophil cytoplasmic antibody-associated vasculitis
AD: Atopic dermatitis
ANA: Anti-nuclear antibody
AU: Arbitrary unit
BCL: B-cell/CLL lymphoma 6
BCOR: BCL6 corepressor
BCORP1: BCL6 corepressor pseudogene 1
CLIA: Chemiluminescent immunoassay
COVID-19: Coronavirus infectious disease 2019
ELISA: Enzyme-linked immunosorbent assay
FDR: False discovery rate
FN: False negative
FP: False positive
GST: Glutathione S-transferase
GTEx: Genotype-Tissue Expression
HC: Healthy control
IFN: Interferon
IgG: Immunoglobulin G
IQR: Interquartile range
KAT2A: K-acetyltransferase 2 A
MCC: Matthews correlation coefficient
ORF: Open reading frame
PCA: Principal component analysis
PBS: Phosphate-buffered saline
PBST: Phosphate-buffered saline with Tween-20
PWAbS: Proteome-wide autoantibody screening
RBD: Receptor-binding domain
ROC: Receiver-operator characteristic
ROC-AUC: Area under the receiver-operator characteristic curve
RT-PCR: Reverse transcription-polymerase chain reaction
SAL: Sum of autoantibody levels
SARS-CoV-2: Severe acute respiratory syndrome coronavirus 2
SLE: Systemic lupus erythematosus
SSc: Systemic sclerosis
SVM: Support vector machine
TBST: Tris-buffered saline with Tween-20
TN: True negative
TP: True positive
TPM: Transcripts per million
UT-ABCD: aUToAntiBody Comprehensive Database
WPA: Wet protein array
XGBoost: Extreme gradient boosting
