# Supplementary material for: Proteome-wide autoantibody screening and holistic autoantigenomic analysis unveil COVID-19 signature of autoantibody landscape
Source: BMC Immunol. 2026 Mar 21;27:52. doi: 10.1186/s12865-026-00826-8 (PMC13321555; doi:10.1186/s12865-026-00826-8)
Supplement: Supplementary file 1 — Supplementary Material 1. [file 12865_2026_826_MOESM1_ESM.docx]

**Extended Figure 1. Distribution of autoantibody levels among different conditions. (A)** A box plot illustrates sum of autoantibody levels (SAL) in each individual by different condition. SAL was defined as the sum of serum levels for all 13,352 autoantibodies tested in our study. ns: P > 0.05, ***: P < 0.001. P-values were calculated by two-sided Mann Whitney U test compared to HCs. **(B)** Box plots show SAL by sex and age groups. P-values were calculated by two-sided Mann Whitney U test compared to HCs. **(C)** A jitter plot displays the distribution of Z-scores for each measured autoantibody in healthy controls (HCs), arranged in descending order of their maximum Z-score values. The blue horizontal dashed line marks Z-score = 3. The red horizontal dashed line marks Z-score = 4. The green horizontal dashed line marks Z-score = 5. **(D)** A histogram illustrates the distribution of Z-scores for all autoantibody measurements in HCs, with a blue vertical dashed line marking Z-score = 3, a red vertical dashed line marking Z-score = 4, and a green vertical dashed line marking Z-score = 5. **(E)** A cumulative histogram illustrates the distribution of Z-scores for all autoantibody measurements in HCs, with a blue vertical dashed line marking Z-score = 3, a red vertical dashed line marking Z-score = 4, and a green vertical dashed line marking Z-score = 5.

**Extended Figure 2. Autoantibodies to cytokines or their receptors. (A)** The heatmap’s columns display the serum autoantibody concentrations targeting cytokines in each subject evaluated by our proteome-wide autoantibody screening. **(B)** The heatmap’s columns display the serum autoantibody concentrations targeting cytokine receptors in each subject evaluated by our proteome-wide autoantibody screening. **(C)** A box plot presents the serum levels of anti-interferon alpha 2 (IFNA2) Abs in the subjects. **(D)** Another box plot indicates the serum levels of anti-interferon alpha 4 (IFN4A) Abs in the subjects.

**Extended Figure 3. Distribution of normalized serum autoantibody levels among different conditions. (A)** A box plot illustrates sum of normalized autoantibody levels in each individual by different condition. Sum of autoantibody levels (SAL) was defined as the sum of normalized serum levels for all 13,352 autoantibodies tested in our study. ns: P > 0.05, ***: P < 0.001. P-values were calculated by two-sided Mann Whitney U test compared to healthy controls (HCs). **(B)** Box plots show normalized SAL by sex and age groups. P-values were calculated by two-sided Mann Whitney U test compared to HCs. **(C)** A histogram illustrates the distribution of normalized values for all autoantibody measurements in HCs.

**Extended Figure 4. Feature importance of top highlighted autoantibodies in other candidate machine learning frameworks.** Autoantibodies a that are mostly highlighted according to feature importance in two-class classification by **(A)** Simple linear regression, **(B)** Ridge regression, **(C)** Logistic regression with normalization, **(D)** Logistic regression with standardization, **(E)** SVM with normalization, and **(F)** SVM with standardization. For simple linear regression, Ridge regression, and logistic regression, the feature importance was indicated by their mean absolute coefficient in the 10-fold cross validation. For SVM, the feature importance is represented by F-scores, meaning the average number of splits by each feature over the 10-fold cross-validation. Autoantibodies and clinical features that are mostly highlighted according to feature importance in two-class **(G)**, three-class **(H)**, and multi-class **(I)** classifications by XGBoost. The feature importance is represented by F-scores, meaning the average number of splits by each feature over the cross-validation.

**Extended Figure 5. Performance of minimal feature models based on XGBoost using the top 1–5 features.** The top-ranked 1–5 features identified by XGBoost, or all the measured autoantibodies, were utilized for a 2-class classification task to discriminate COVID-19 cases.

**Extended Figure 6. Serum levels of anti-BCORP1 and anti-KAT2A Abs by sex and age. (A)** A scatter plot depicts the correlation between serum levels of anti-KAT2A autoantibodies measured by wet protein arrays (WPAs) and enzyme-linked immunosorbent assays (ELISA). The red line represents the regression line, while the gray shaded area denotes the 95% confidence interval. AU: arbitrary unit. **(B)** A scatter plot depicts the correlation between serum levels of anti-BCORP1 autoantibodies measured by WPAs and serum levels of anti-BCOR autoantibodies by ELISA. The red line represents the regression line, while the gray shaded area denotes the 95% confidence interval. AU: arbitrary unit. **(C)** Serum levels of anti-BCORP1 Abs by sex and age groups. ns: P > 0.05, *: P < 0.05, **: P < 0.01, ***: P < 0.001. P-values were calculated by two-sided Mann Whitney U test compared to HCs. Red vertical dashed lines indicate Z-scores = 4 in HC. **(D)** Serum levels of anti-KAT2A Abs by sex and age groups. ns: P > 0.05, *: P < 0.05, **: P < 0.01, ***: P < 0.001. P-values were calculated by two-sided Mann Whitney U test compared to HCs. Red vertical dashed lines indicate Z score = 4 in HC.
