# Supplementary figures and images for: Proteome-wide autoantibody screening and holistic autoantigenomic analysis unveil COVID-19 signature of autoantibody landscape
Source: BMC Immunol. 2026 Mar 21;27:52. doi: 10.1186/s12865-026-00826-8 (PMC13321555; doi:10.1186/s12865-026-00826-8)

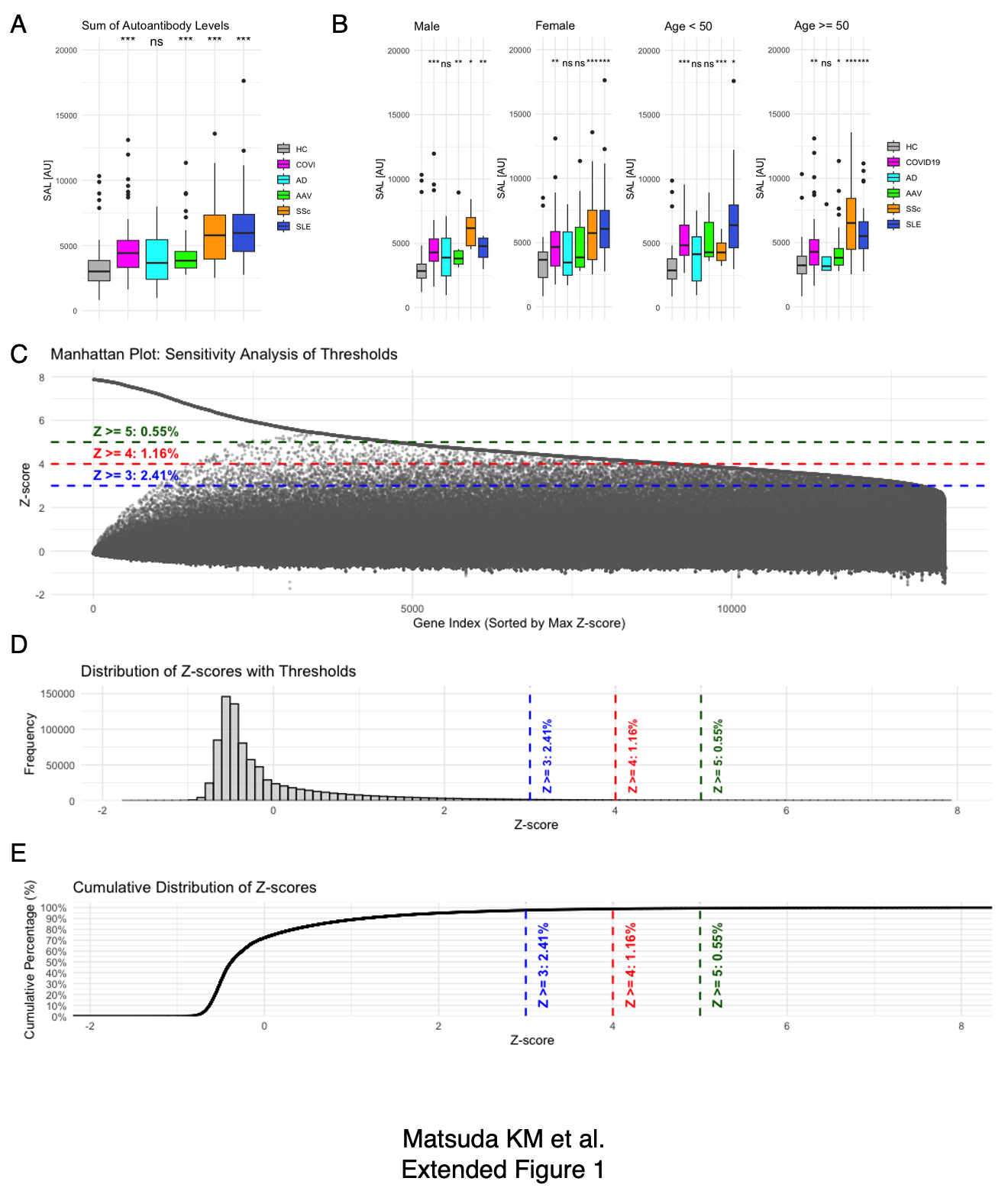

Supplement: Supplementary file 2 — Supplementary Material 2. [file 12865_2026_826_MOESM2_ESM.tiff]

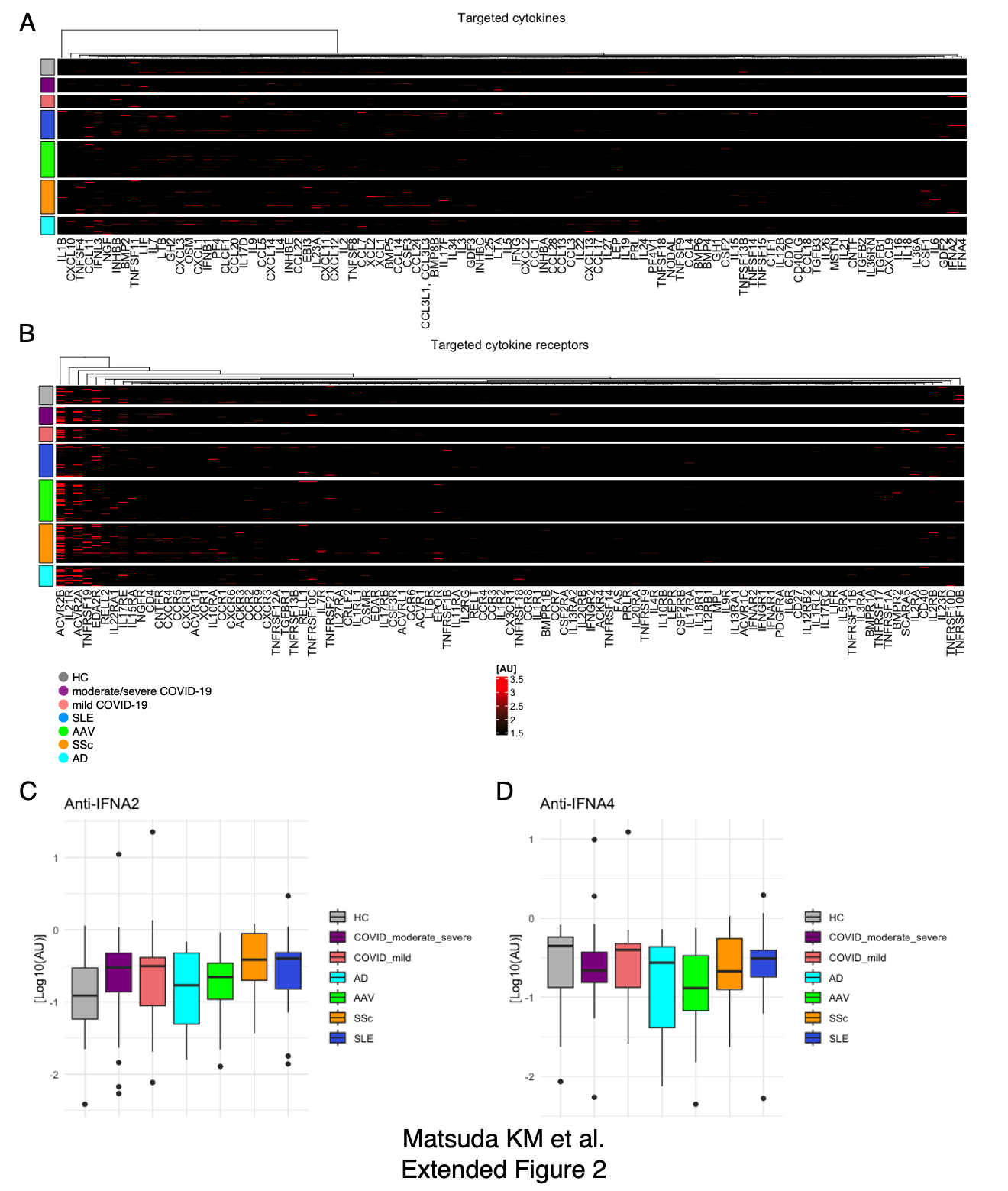

Supplement: Supplementary file 3 — Supplementary Material 3. [file 12865_2026_826_MOESM3_ESM.tiff]

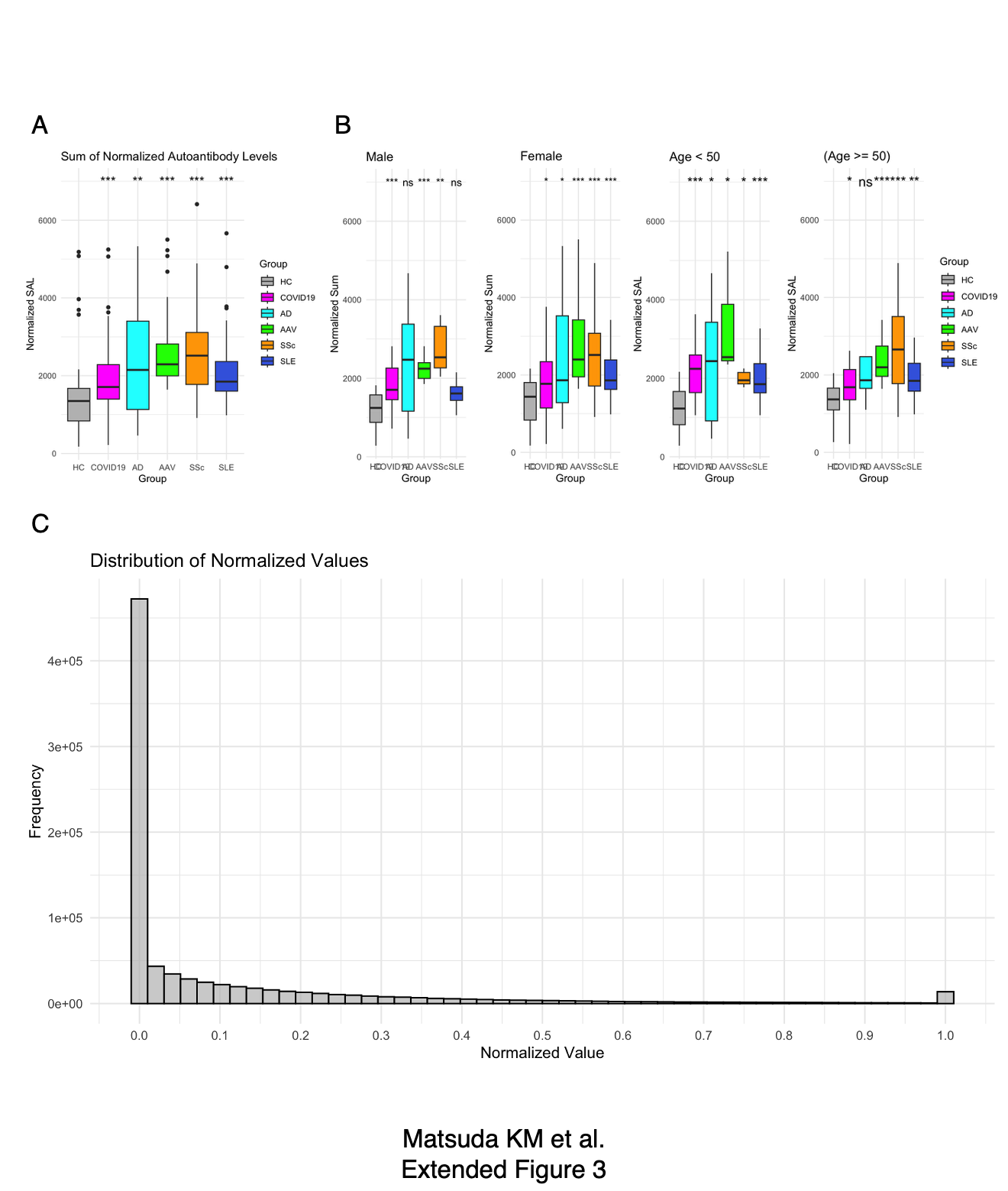

Supplement: Supplementary file 4 — Supplementary Material 4. [file 12865_2026_826_MOESM4_ESM.tiff]

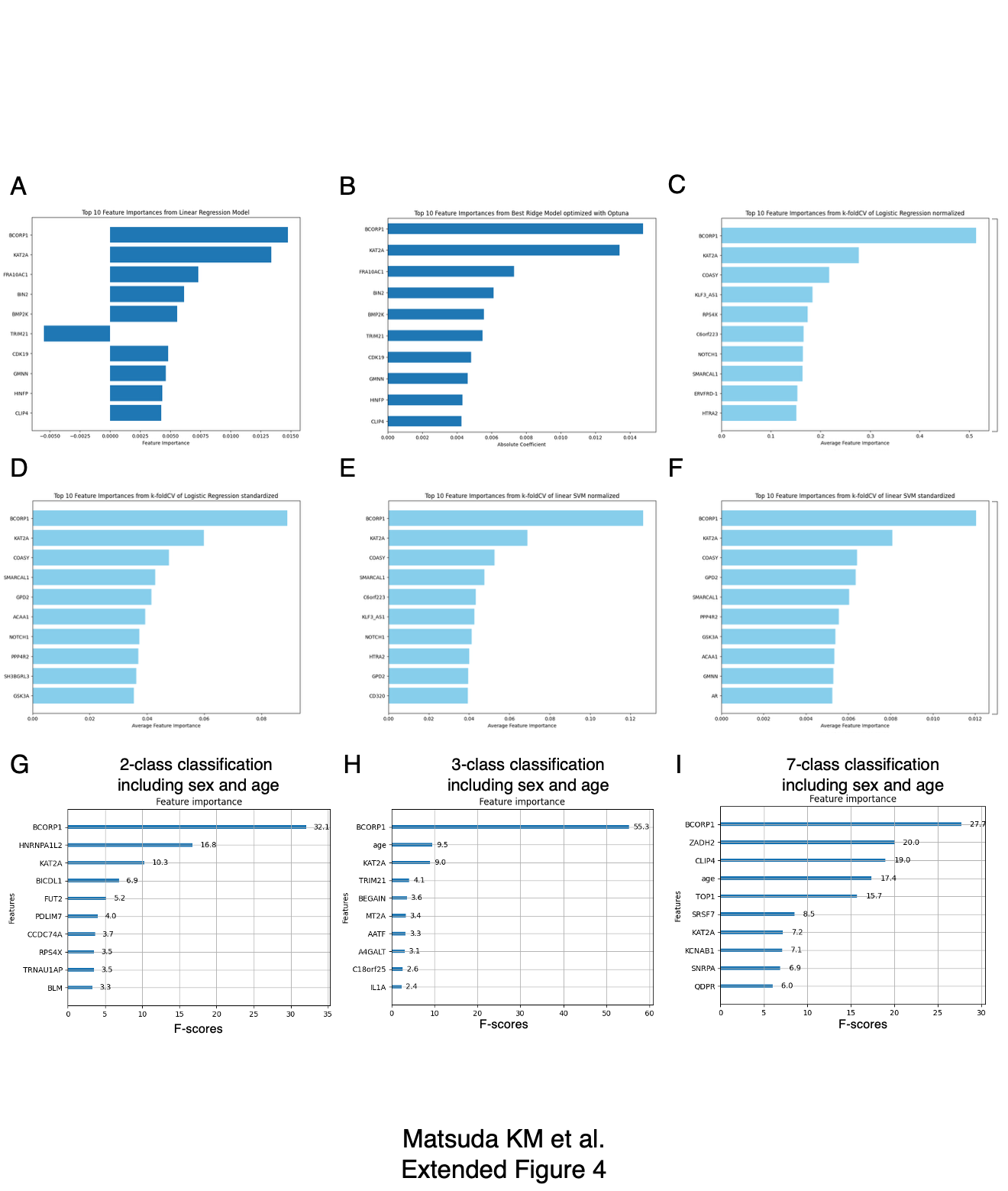

Supplement: Supplementary file 5 — Supplementary Material 5. [file 12865_2026_826_MOESM5_ESM.tiff]

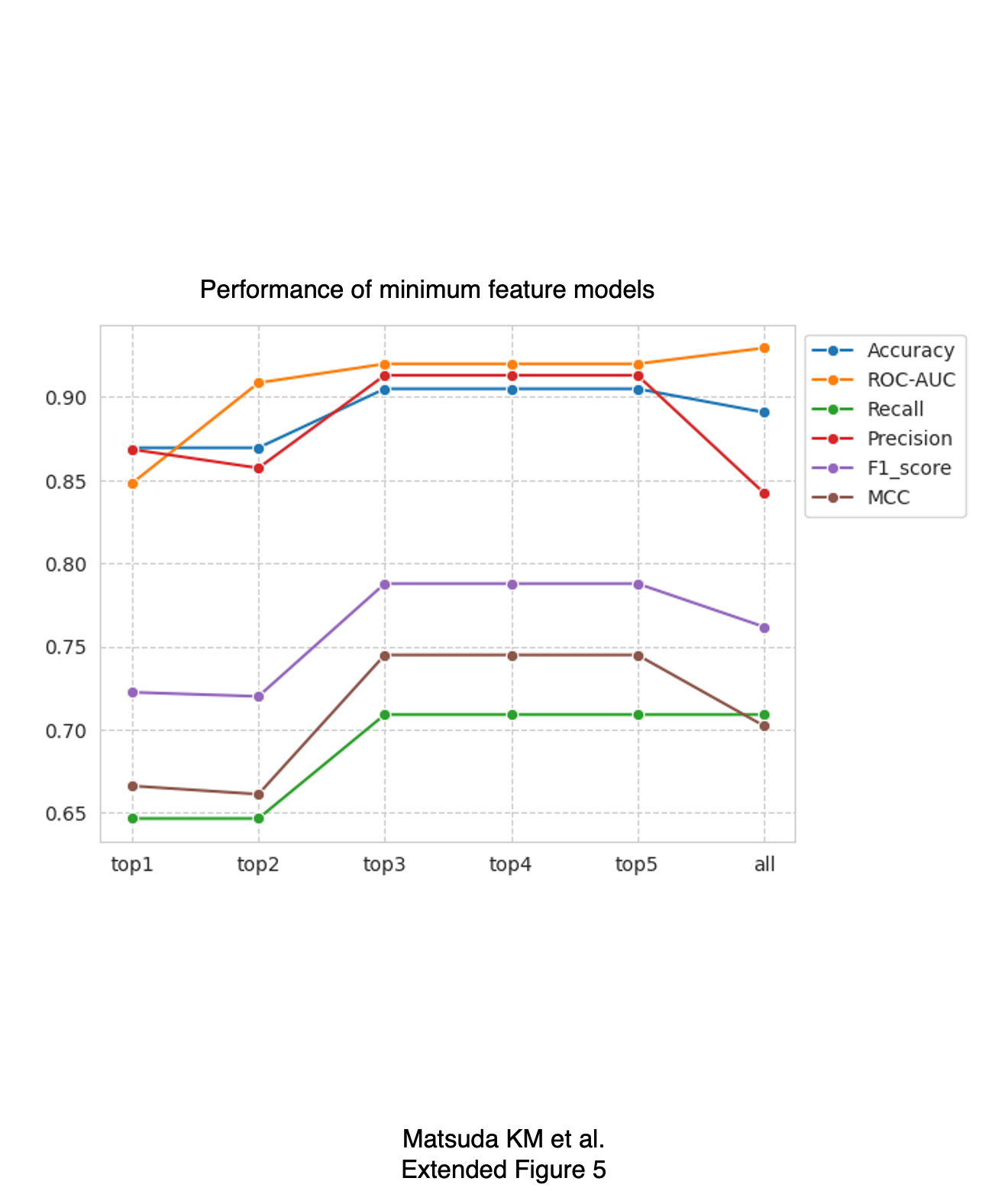

Supplement: Supplementary file 6 — Supplementary Material 6. [file 12865_2026_826_MOESM6_ESM.tiff]

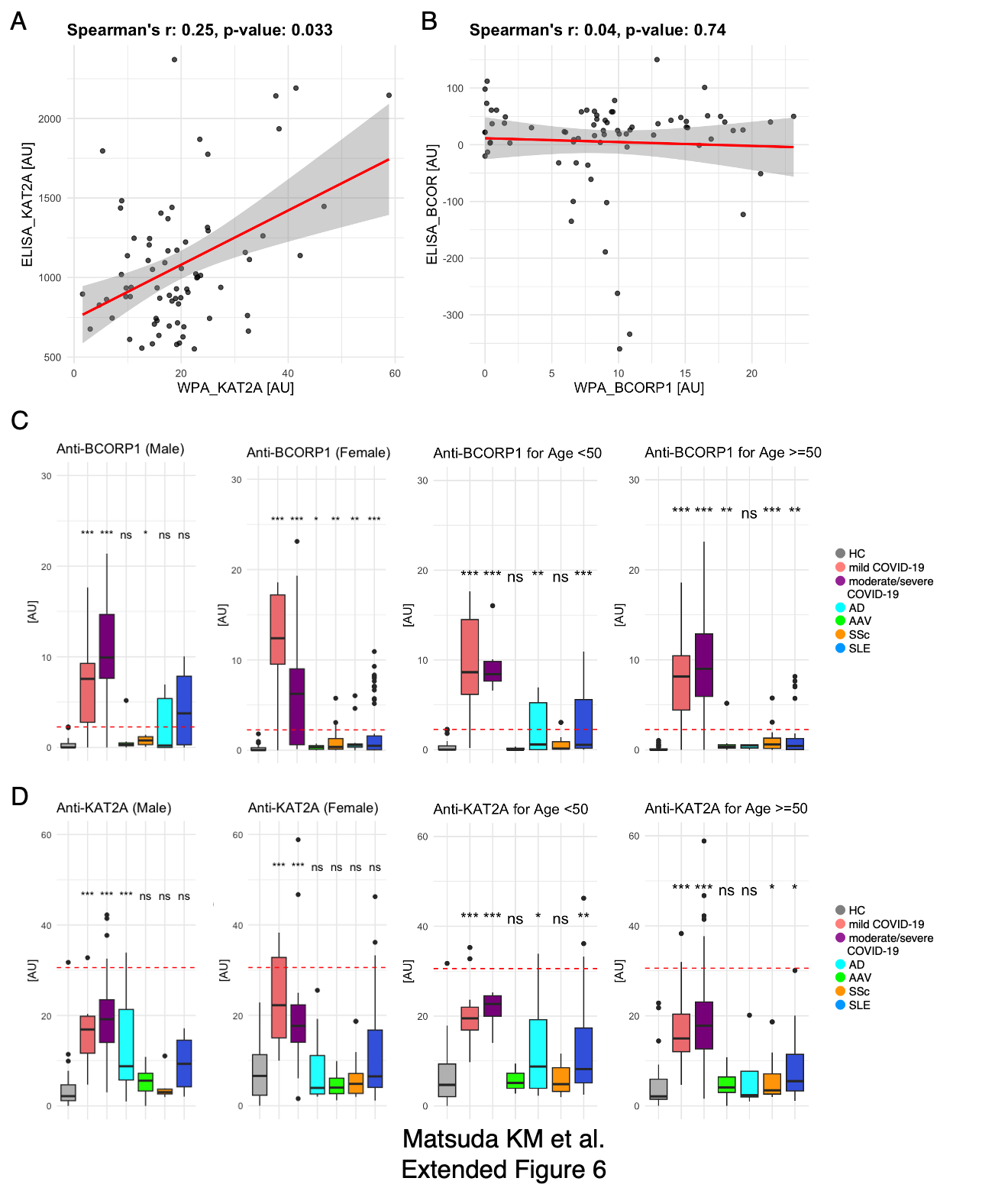

Supplement: Supplementary file 7 — Supplementary Material 7. [file 12865_2026_826_MOESM7_ESM.tiff]
